# Acute Delirium Post-inguinal Hernia Mesh Repair in a 40-Year-Old Male: An Unusual Case of Cystocerebral Syndrome

**DOI:** 10.7759/cureus.42260

**Published:** 2023-07-21

**Authors:** Gagandeep Singh Arora, Parneet Kaur

**Affiliations:** 1 Internal Medicine, University of California Riverside, San Bernardino, USA; 2 General Surgery, Government Medical College, Patiala, Patiala, IND; 3 Emergency Department, Civil Hospital Mukerian, Mukerian, IND; 4 Internal Medicine, Suburban Community Hospital, Philadelphia, USA

**Keywords:** inguinal hernia mesh repair, spinal anesthesia, elderly patient, bladder overdistension, agitation, acute urinary retention, cystocerebral syndrome, postoperative complications, delirium, urinary retention

## Abstract

Acute urinary retention is a known complication of inguinal hernia repair. However, the development of severe agitation and delirium as a result of acute urinary retention following inguinal hernia repair is less commonly reported. Here, we present the case of a 40-year-old male with no relevant medical history who underwent open mesh hernia repair for an uncomplicated left-sided indirect inguinal hernia. Postoperatively, the patient became hypertensive, delirious, and violent. He was found to have urinary retention on a bladder scan. Urgent intervention with catheterization and bladder decompression resulted in the prompt resolution of the patient’s symptoms. The patient regained his senses and did not remember the events that led to it. This case highlights the importance of recognizing and managing acute urinary retention to prevent the development of severe agitation and delirium following spinal anesthesia. Further research and awareness are necessary to better understand the underlying neurovisceral mechanisms and optimize preventive strategies.

## Introduction

Delirium is a complex neuropsychiatric syndrome characterized by acute cognitive impairment and fluctuating attention [1]. It is a common and serious complication, particularly in elderly and medically complex patients [1]. While various factors (medical conditions, medications, surgery, sleep deprivation, environmental factors, emotional stress, dehydration, pre-existing cognitive impairment, electrolyte imbalances, and infections) can contribute to the development of delirium, postoperative patients are particularly susceptible due to the interplay of surgical stress, anesthesia, and physiological changes [1]. Among the numerous etiological factors, urinary retention can also cause delirium [2].

Urinary retention, characterized by impaired emptying of the bladder, can occur as a consequence of surgical procedures, including hernia repair surgeries [3]. In such cases, the mechanical manipulation of tissues, the effects of anesthesia, and the use of analgesics can disrupt the normal functioning of the bladder [4]. If left unrecognized or untreated, urinary retention can lead to bladder distension, urinary stasis, and subsequent complications [5].

Studies have shown that bladder distension and urinary retention can precipitate delirium, particularly in vulnerable individuals [2]. This phenomenon, known as cystocerebral syndrome [2], is believed to be mediated by sympathetic overdrive secondary to the distended bladder leading to cognitive dysfunction and the development of delirium [6]. Understanding the pathophysiological interplay between urinary retention and delirium is crucial for early recognition and intervention, as prompt management of urinary retention can prevent or alleviate delirium symptoms [6].

In this report, we present the case of a 40-year-old male who developed acute delirium following inguinal hernia repair, secondary to postoperative urinary retention (POUR) causing bladder distension. We aim to highlight the importance of identifying and promptly managing urinary retention as a potential cause of delirium, emphasizing the need for healthcare providers to be vigilant in their assessment and monitoring of postoperative patients. Furthermore, we discuss the challenges associated with the use of Foley catheterization as a management strategy, as it carries potential risks such as increased postoperative stay and catheter-associated urinary tract infections.

By elucidating the connection between surgery, urinary retention, and delirium, this case report underscores the importance of early recognition and targeted management strategies to prevent the development of urinary retention delirium and promote successful surgical outcomes.

## Case presentation

A 40-year-old male with no relevant past medical history presented to our outpatient clinic with complaints of dragging pain in the inguinal region. Additionally, the patient had no past history of seizure disorder or recreational drug abuse and denied any symptoms of benign prostatic hyperplasia or urinary difficulties. He was a lifetime non-drinker and non-smoker.

Upon physical examination, a noticeable swelling was seen located just above the left inguinal ligament. Palpation confirmed the presence of a soft, reducible non-tender mass in the left inguinal canal arising from the deep inguinal ring. The hernia became more prominent with coughing. The testicular examination was normal. Bowel sounds were normal. These findings were consistent with a diagnosis of left-sided indirect inguinal hernia. The patient reported that the symptoms had gradually worsened over the years, but he had delayed seeking medical attention until the pain became unbearable.

Preoperative assessment

Before the planned inguinal hernia mesh repair surgery, a comprehensive preoperative evaluation was performed. The patient underwent routine blood work investigations, including a complete blood count, comprehensive metabolic panel, and complete urine examination. Notably, all preoperative lab results were within the normal range, ruling out any underlying systemic abnormalities or urinary infections.

Surgical procedure

The patient was scheduled for an open mesh hernia repair surgery. Under spinal anesthesia, the procedure was performed without any complications. The hernial sac was dissected, the contents of the sac were reduced with ease, and the sac was transfixed with a vicryl 3-0 on the round body. The defect was meticulously closed, and a prolene mesh was placed to provide optimal reinforcement after achieving hemostasis. Closure of the skin incision was done as usual with nylon 2-0 on the cutting needle.

Postoperative course

Following the surgery, the patient was transferred to the post-anesthesia care unit (PACU). He initially passed a small amount of urine, indicating some degree of normal bladder function, remained conscious, and his vital signs, including oxygen saturation, remained stable within normal limits. He was following commands and was able to hold a conversation. After an hour, he was shifted to the ward.

Development of acute delirium

While in the ward, the patient was being given pain relief with intravenous (IV) Tylenol. He confirmed being pain-free while in the ward. Approximately two hours after being admitted to the ward, the patient started experiencing agitation, along with a feeling of heaviness in the head and discomfort. These symptoms progressively worsened, and he became unresponsive and delirious. His blood pressure gradually increased, reaching 170/100 mmHg, despite no history of hypertension. The patient’s initial assessment revealed a Glasgow Coma Scale score of 7 (E1 M4 V2), indicating minimal eye-opening (1), withdrawal from pain motor response (4), and incomprehensible verbal response (2). Additionally, the Richmond Agitation-Sedation Scale was measured at plus 4, denoting a state of overt combative agitation.

Prompt evaluation and management

**Figure 1:**
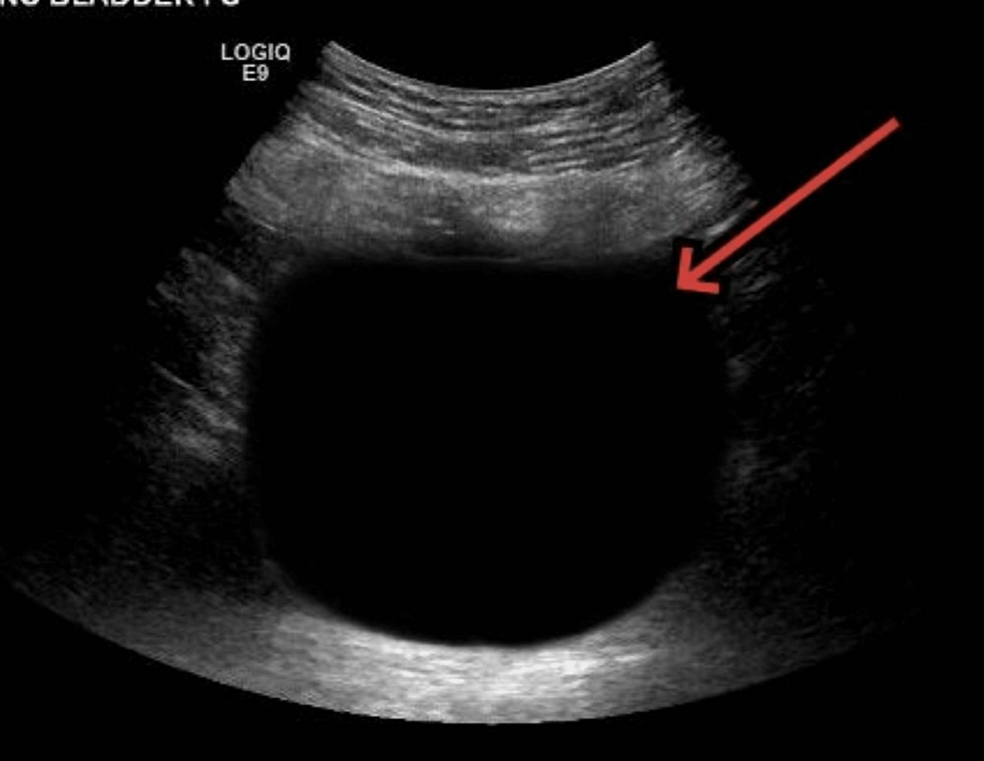
Bedside ultrasound image of the urinary bladder. The red arrow points toward the distended urinary bladder.

Given the patient’s worsening clinical condition, an urgent assessment of the operative site was performed to rule out any active bleeding or surgical complications. However, no abnormalities were observed and the surgical site appeared healthy. His pupils were bilaterally equal and reactive to light. At this point, he became violent and was pulling IV lines, so we decided to administer 1 mg of IV Ativan, which failed to calm him down. Labs were drawn immediately to look for electrolyte abnormalities. Abdominal palpation revealed mild bladder distention. To evaluate the bladder volume, a bedside ultrasound was conducted, revealing a bladder capacity of 600 mL (Figure 1).

Urinary retention and catheterization

Based on the findings of significant bladder distention, a diagnosis of urinary retention was established. With the patient exhibiting extreme agitation and movement, urinary tract catheterization was challenging but successfully performed. A total of 600 mL of urine was drained, leading to a rapid improvement in the patient’s consciousness and a remarkable resolution of the delirium. Subsequent blood pressure readings showed a normalization to 120/80 mmHg.

Follow-up head CT and blood investigations

Once the patient regained consciousness and his vital signs stabilized, a follow-up CT scan was conducted to further evaluate the postoperative status. The CT scan was unremarkable, showing no evidence of intracranial abnormalities or other significant findings. Later, blood investigation reports also came back normal.

## Discussion

POUR is the inability to urinate after surgery despite having a full bladder [3], which can cause significant patient distress, discomfort, pain, and increased risk of urinary tract infections [3]. Moreover, if left untreated, POUR can lead to bladder distension, potentially resulting in bladder and kidney damage [3]. Its prevalence ranges from 5% to 70%, making it a common diagnosis in postoperative settings [3].

POUR is a well-recognized complication of inguinal hernia repair [7]. Factors such as old age, diabetes mellitus history, antispasmolytic use, long operation and anesthesia duration, excessive postoperative fluid replacement, and the absence of perioperative non-steroidal anti-inflammatory drug usage have been identified as contributing to the risk of POUR after inguinal hernia repair [4].

One might think why not routinely use Foley catheterization before, during, or after surgery? It is because catheterization has its own risks [8-10]. Sullivan et al. reported bacteremia in over 8% of patients after single catheterization [8]. Dobbs et al. reported that even intermittent catheterization with a straight catheter had similar risks as urinary tract infections [9]. Further, a higher mortality rate has been reported in hospitalized patients who developed nosocomial urinary tract infections after indwelling bladder catheterization [10].

The type of anesthesia is also correlated with the incidence of POUR. Li et al. found a correlation between spinal anesthesia and a higher frequency of POUR compared to general anesthesia [11].

Similar to POUR, postoperative delirium is yet another complication in the immediate postoperative period. Delirium by definition is a neurocognitive syndrome caused by reversible neuronal disruption due to an underlying systemic perturbation [1]. Postoperative delirium is commonly recognized in the PACU as a sudden, fluctuating, and usually reversible disturbance of mental status with a degree of inattention [1]. Reduced arousal due to deep sedation can be confused with alterations in brain function, as hypoactive delirium is the most common form of postoperative delirium [1]. The causes of postoperative delirium include exacerbation of a primary injury or insult, potential new secondary insult or injury, substance intoxication delirium, substance withdrawal delirium, medication-induced delirium, delirium because of other medical conditions, delirium because of other etiologies, smoking, intraoperative factors such hypotension and shock, postoperative factors such as pain and sleep-wake disturbances, and alcohol and benzodiazepine withdrawal [1].

Our case discusses a scenario where POUR caused postoperative delirium. This is where understanding cystocerebral syndrome is important.

Cystocerebral syndrome is a term used to describe a state of encephalopathy or delirium characterized by symptoms such as agitation, paranoia, confusion, reduced responsiveness, and difficulty with redirection which typically occurs in the presence of bladder distention [2]. The syndrome has been classically described in male patients older than 70 years who often had a history of benign prostatic hypertrophy (BPH) [12]. Blackburn and Dunn in 1990 proposed the concept of cystocerebral syndrome, where acute urinary retention in elderly patients could present as sudden confusion or delirium [2]. These symptoms often rapidly resolve upon bladder decompression [2].

Further exploration into the neurophysiological mechanisms was done by Shirvani et al., who attributed the confusion and agitation associated with bladder distention to increased sympathetic tone and consequent catecholamine release [6]. This is especially relevant in individuals with underlying cognitive deficits [6]. Unnoticed accumulation of urine in the bladder and subsequent distension causes a sympathetic overdrive response leading to delirium [6].

Rickenbacher et al. also underscored the neural circuits linking the brain and viscera, noting that pathologies in the viscera, such as bladder obstruction, could have significant neurobehavioral consequences [13]. Our case echoes the assertion of Rickenbacher et al. on the significance of neurovisceral communication and how disruptions can lead to acute syndromes such as cystocerebral syndrome.

Waardenburg et al. highlighted the prevalence of urinary retention in elderly patients, noting that old age, use of anticholinergics, long-standing diabetes mellitus, and constipation are risk factors [14]. The study further emphasized the importance of immediate bladder decompression in treating such patients [14].

A comprehensive evaluation of patients is recommended in patients with suspected cystocerebral syndrome which includes reviewing the patient’s medical history, evaluating for conditions such as BPH, diabetes mellitus, and constipation, as well as performing necessary physical examinations [12]. Treatment is usually done with immediate bladder decompression and medical management with alpha-adrenergic receptor antagonists such as tamsulosin [12]. Hence, bladder distention is central to the mechanism of cystocerebral syndrome.

Pflug et al. noted the sequence of return of neurological activity after subarachnoid block anesthesia and found that sympathetic activity returns first, then pinprick sensation, followed by somatic motor muscle strength, and, lastly, proprioception in the feet [15]. As the act of micturition falls under motor control, it is regained last [15]. This order of return of neurological functions post-spinal anesthesia further emphasizes the need for vigilance regarding bladder function in the postoperative period. This sequence can shed light on why cystocerebral syndrome might occur even in younger patients postoperatively.

Although the effect of spinal anesthesia on detrusor function has been well-documented, our case accentuates how this effect can potentially predispose even younger individuals to complications such as acute urinary retention, leading to bladder distension [11]. Thus, we can infer that a delay in the return of detrusor function post-spinal anesthesia and early return of sympathetic tone can lead to a state of adrenergic overdrive due to bladder distension, as reported by Shirvani et al. [6]. 

In our case, the patient had no history of alcohol or substance use. His preoperative and postoperative blood investigations including electrolytes were within normal limits. Moreover, our patient developed delirium in the ward and not in the PACU. He was well awake and following commands in the PACU. As we see from the causes of postoperative delirium listed above, urinary retention is not a well-known cause of delirium.

Our case is unique as the patient was only 40 years old and had no past history or symptoms of BPH. The fact that he went into acute delirium three hours after the procedure made everyone concerned about the cause of delirium. He had become severely agitated and was difficult to calm down. His blood pressure had likely shot up because of adrenergic overdrive. We could not do a head CT because he was not lying still. Hence, it is important to recognize that the patient may have come out of the sedation given during the procedure but the visceral organs take some time to regain their sensation.

**Figure 2:**
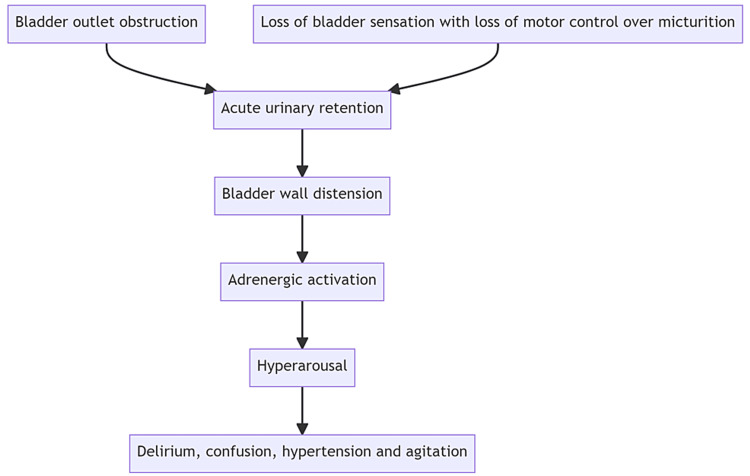
Flow diagram showing the proposed mechanism of delirium and agitation (cystocerebral syndrome). In our patient, there was no bladder outlet obstruction but bladder distension occurred because of loss of bladder sensation and motor control over micturition. Flowchart made at mermaid.live.

This case should help us understand that distension of the bladder due to any cause can lead to an adrenergic overdrive and cystocerebral syndrome. A flowchart illustrating this proposed mechanism is shown in Figure 2.

Although it is classically described in elderly patients with BPH, it can also occur in the absence of a mechanical obstruction where the patient does not have a sensation of his bladder in the postoperative period.

## Conclusions

Cystocerebral syndrome, traditionally associated with elderly individuals, can also present in young patients, especially when they are subjected to procedures under spinal anesthesia. The delay in the return of bladder sensation and function after spinal anesthesia could pave the way for bladder distention and acute urinary retention, precipitating this syndrome. It emphasizes the importance of vigilant monitoring of urinary function in patients of all ages following surgeries under spinal anesthesia. This can aid in the early recognition and management of urinary retention, potentially mitigating the development of cystocerebral syndrome and improving patient outcomes. This case underlines the need for an improved understanding and awareness of the intricate link between urinary and neurological systems, necessitating the refinement of strategies to prevent and manage such postoperative complications.
